# Psychophysiological research in real-world environments: methodological perspectives from the SLU Multisensory Outdoor Laboratory

**DOI:** 10.3389/fpsyg.2025.1432180

**Published:** 2025-05-30

**Authors:** Gunnar Cerwén, Caroline M. Hägerhäll

**Affiliations:** ^1^SLU Multisensory Outdoor Laboratory (SENSOLA), Swedish University of Agricultural Sciences, Alnarp, Sweden; ^2^Department of Landscape Architecture, Planning and Management, Swedish University of Agricultural Sciences, Alnarp, Sweden; ^3^Department of People and Society, Swedish University of Agricultural Sciences, Alnarp, Sweden

**Keywords:** psychophysiology *in situ*, wearable technology, environmental psychology, landscape architecture, skin conductance, heart rate variability, eye tracking, fNIRS

## Abstract

Growing evidence demonstrates the importance of environmental quality for human health and wellbeing. Environmental psychology can inform planning and design of future environments, but previous research often used simulated settings, limiting ecological validity. To bridge this gap and enable studies in real environments, a new laboratory (SENSOLA) has been built at the Swedish University of Agricultural Sciences. The laboratory is designed to facilitate studies on human-environment interactions in real environments, with a particular focus on psychophysiology using wearable sensors. An important prerequisite of the endeavor is the ability to synchronize environmental data with biomarkers and participants’ self-reports over time. In this methodological paper, we describe the creation and implementation of the SENSOLA laboratory. Drawing on experiences gained from the first ten studies conducted within the laboratory, we summarize key considerations for conducting research in field settings. We discuss various methodological approaches and procedural considerations, highlighting challenges and possibilities, to serve as a peer-reviewed guideline for future studies in the lab and elsewhere.

## 1 Introduction

A growing body of research in environmental psychology and public health highlights the importance of high-quality outdoor environments for human health and wellbeing (WHO, 2003; Rao et al., 2007). The environment has far-reaching effects on everyday life, influencing behavior and social interaction (Gibson, 1979; Costall, 1995; Steg and Vlek, 2009; Brown and Lombard, 2014), preference (Brady and Prior, 2020), attention and cognitive functioning (Kaplan and Kaplan, 1989; Berman et al., 2008; Bratman et al., 2012), stress (Ulrich, 1983; Yao et al., 2021), as well as restoration and resilience (Kaplan and Kaplan, 1989; Weber and Trojan, 2018; White et al., 2023) among other things. To understand the underlying mechanisms of these effects, psychophysiological methods provide a valuable tool for measurement complementing self-reported experiences and observations. While some effects can be studied in laboratory settings, others can only be examined in real-world environments.

Environmental psychology emerged as a somewhat fragmented field of research, with early 20th century roots in studies on work performance, architecture and moral philosophy (Gifford et al., 2011). By mid-century, the field had established itself with research on topics like “sensory isolation, personal space, and building design” (Gifford et al., 2011). A central idea was to understand the interplay between human psychology and physical environments, and to do this in real-world settings (Steg et al., 2019).

However, a significant portion of subsequent research in the field instead relied on representations of outdoor environments in controlled laboratory settings, offering control over stimuli and convenient participant sampling. Additionally, the laboratory facilitated the use of physiological markers (see, e.g., Ulrich et al., 1991), with equipment originally designed for medical purposes. Inspiration likely came from related fields like psychophysiology (Stern et al., 2001d) and neuroscience (McCunn, 2024).

Laboratory studies offer control. Researchers can pinpoint specific environmental aspects by manipulating stimuli, typically using images, videos, and sounds in various combinations. While confounding factors are minimized, the ecological validity, or real-world relevance, of these studies is questionable as the stimuli are merely representations of the actual environment (c.f. Houtveen and de Geus, 2009). As Rohrbaugh (2016) points out, simulations may miss crucial aspects of the complexity experienced in everyday life.

In the study of scenic beauty and landscape preference, photographs were early on reported as valid substitutes for real environments (Shuttleworth, 1980; Nassauer, 1983; Zube et al., 1987; Kaplan and Kaplan, 1989) and was favored for the advantage of being able to include larger numbers of both participants and different settings. Voices questioning the validity of photo-based studies were however also heard, pointing at for instance the impact of purpose and goal directed behavior on scenic beauty estimations when engaged in recreation in real settings (Hull and Stewart, 1992).

Moreover, natural interaction with an environment is arguably impossible to simulate when a participant knows they are in a controlled laboratory. Even though simulation can be accomplished with impressive accuracy in virtual reality (Parsons, 2015), augmented reality (Berryman, 2012), or large scale laboratories (Vigliocco et al., 2024), the mere knowledge of participating in a controlled experiment changes how people interact with the environment. For instance, when reporting on their experiences on eye tracking, Uttley et al. (2018) explain how participants in a laboratory setting tend to look more at people compared with participants using mobile eye tracking. An explanation for this difference would be that people worry about the reactions that could follow when looking at someone in real life, which of course is not an issue when looking at a person in a video.

The technological development has gradually increased the possibility to study environmental experiences *in situ*. While the earliest initiatives for ambulatory measurement systems were developed for clinical use (Rohrbaugh, 2016), subsequent development has been driven also by the consumer oriented market and the increasing use of smart technology. Various devices for health monitoring, such as smart watches, health rings and mobile applications continue to be presented with a rapid pace.

Consequently, an increasing number of studies have been published that incorporates wearable sensors in various ways; topics include assessment and reviews of specific wearable devices (Shcherbina et al., 2017; Peake et al., 2018), comparisons between technologies and procedures (Haynes and Yoshioka, 2007; Castaneda et al., 2018), experiments where participants are sitting in outdoor environments (Horiuchi et al., 2014), walking (Gidlow et al., 2016), sitting and walking (Korpilo et al., 2024) or traveling by bike (Marquart et al., 2022) or car (Anciaes, 2023). Studies of everyday life may also involve diaries, questionnaires and other forms of self-reports, sometimes in combination with psychophysiological measurements and monitoring of physical activity and body movement (Wilhelm et al., 2012). Additionally, wearable technology is increasingly used to facilitate self-reports of environmental experiences with the aid of smart phone applications or padlets, so called experience sampling or ecological momentary assessment (Beute et al., 2016).

The shift toward studies *in situ* could offer important new revelations about people-environment interaction. However, studies in situ poses several new challenges that require consideration, including data artefacts, confounding factors and natural variations in the environment. A key question is how we can incorporate recurring events that are actually an important part of the environment and distinguish them from singular events that would be a true confounder. Furthermore, a continuous recording of data, capturing the experience as it unfolds, is fundamentally different from a traditional controlled experiment with a stimulus – response design. This requires new approaches to the data analyses and interpretation of results. The present paper summarizes the work and experiences carried out at the SLU Multisensory Outdoor Laboratory, SENSOLA, sharing our perspectives and proposing a guideline for the future design of studies in situ.

## 2 Background

### 2.1 SENSOLA: a research infrastructure with possibility to study human-environment interactions *in situ*

SENSOLA is a research infrastructure project financed by the Swedish University of Agricultural Sciences. The lab mainly operates at the intersection of environmental psychology and landscape architecture, with the mission of expanding empirical and methodological knowledge on human-environment interactions in outdoor settings. A key objective for the lab is to increase ecological validity by taking research into actual environments. To achieve this, the lab holds a set of portable equipment for studies in situ as well as a complementary section for controlled studies indoors (for further details, see section 3).

It is known that physiology can be used to infer important aspects of human experience – such as arousal, attention, emotional state, and cognitive engagement – providing insights into how individuals interact with and are affected by their environments (Bradley and Lang, 2000; Parsons and Tassinary, 2002; Dawson et al., 2016; Berntson et al., 2017; Ancora et al., 2022). While research in controlled settings has demonstrated that physiological measures, including electrodermal activity, cardiovascular responses, and neuroimaging, can provide valuable, objective data on human responses (see, e.g., Ulrich et al., 1991; Parsons et al., 1998; Hagerhall et al., 2008; Annerstedt et al., 2013), the feasibility and validity of these measures in dynamic, real-world environments remain insufficiently explored. By conducting research *in situ*, SENSOLA aims to investigate how these measures are influenced by, and how they can be used to understand, the complexities of outdoor environments. This involves integrating objective data from wearable sensors with self-reports and observational data to gain a deeper understanding of how different environmental factors influence human well-being, preference, and behavior. Research conducted within the SENSOLA lab is expected to have significant implications for landscape design by providing objective evidence of how people physiologically respond to different design elements. For example, data on arousal levels can help assess the stimulating or calming effects of different spaces, while data on attention and cognitive engagement can inform the design of spaces that minimize distractions and promote focus.

#### 2.1.1 Lab building process

**Figure 1:**
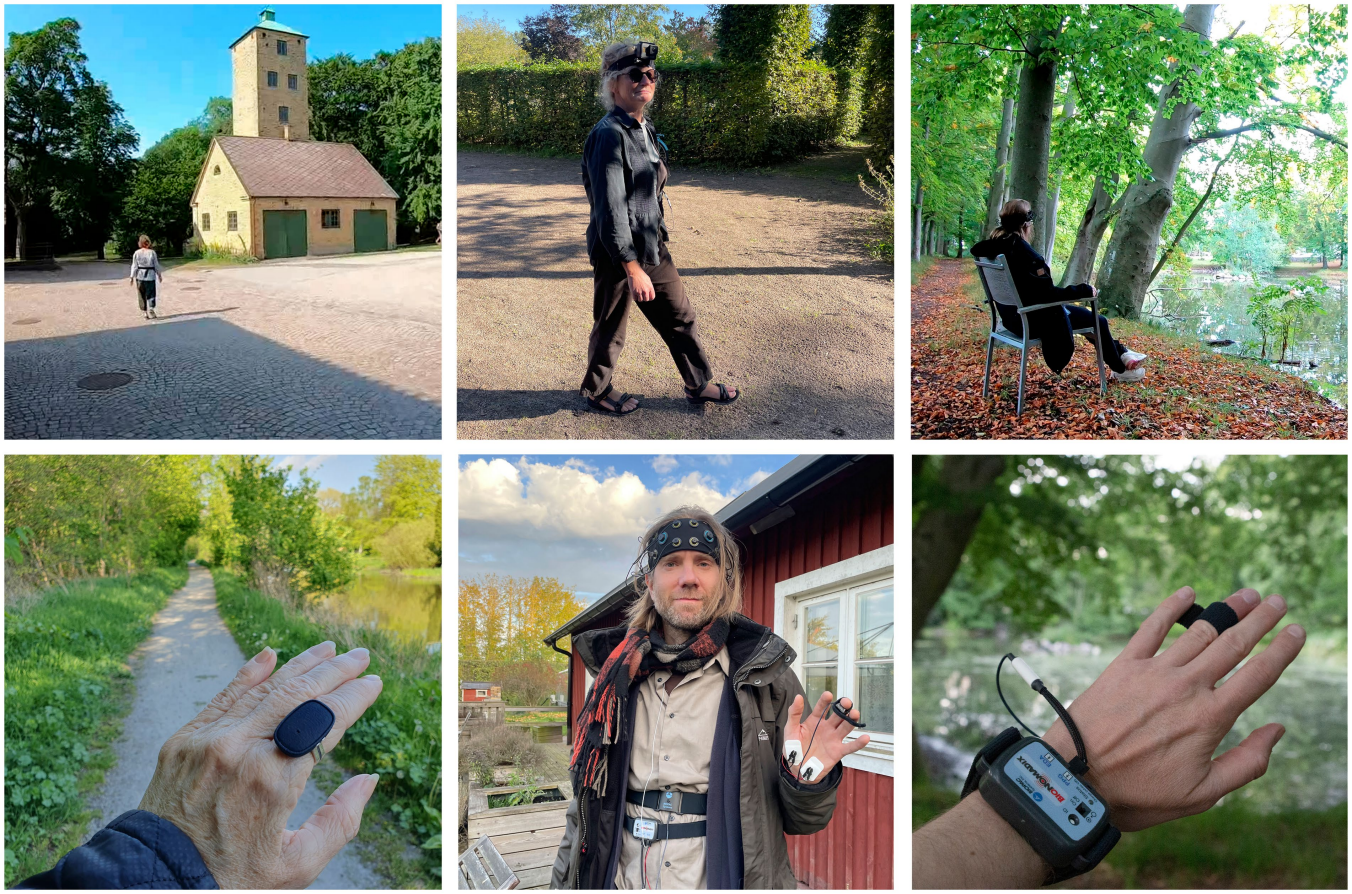
Documentation of pilot studies and equipment testing. Top left: SENSOLA Pilot 1. Top center: SENSOLA pilot 2. Top right: SENSOLA pilot 4. Bottom left: SENSOLA pilot 6. Bottom center: Synchronization test. Bottom right: Equipment test.

To establish the lab, a postdoctoral researcher served as the lab coordinator, working closely with the project leader to oversee system integration. An internal project group and an external advisory board, selected for their expertise relevant to the initial project proposal, also provided guidance. We examined existing laboratories, evaluated technologies, and contacted manufacturers to identify suitable equipment, considering factors like intrusiveness, data security, and synchronization capabilities. A focus was placed on avoiding subscription plans to ensure long-term lab flexibility. Equipment was evaluated as an integral part of the lab building process, through testing during pilot studies and other exploratory research activities (See Figure 1 and Supplementary Appendix 1).

The laboratory has been actively promoted through seminars and events, where insights into the lab’s development process and pilot study experiences are shared with both university and external stakeholders. SENSOLA is currently available as a research infrastructure to support a wide array of projects, with a focus on transdisciplinary research in environmental psychology and landscape architecture. Operating in a field characterized by constant advancements in products and solutions, the lab is not a static endeavor but will continue to develop in new directions.

#### 2.1.2 Pilot studies

Ten initial studies were conducted concurrently with the lab development. While not all specifically designed to inform lab development, these studies provided valuable insights into conducting psychophysiological research *in situ* (for further details on the studies, see Supplementary Appendix 1). These pilot studies yielded valuable methodological takeaways, including: (a) an increased understanding regarding the general feasibility of conducting psychophysiology in situ (b) pragmatic and logistical considerations on procedure (c) improved understanding of how to prepare and select a study site (d) strategies for addressing confounding factors (e) the feasibility of using different physiological markers on moving subjects.

In the coming sections of the paper, we discuss our work in relation to previous research and outline our perspectives as a guideline for future psychophysiological studies in situ.

### 2.2 Psychophysiological data

In this section, we briefly describe some of the basic physiological markers we use in the lab and how they relate to psychological processes. For a deeper description of the markers and suggestions for data treatment strategies, see Supplementary Appendix 2.

#### 2.2.1 Cardiovascular system

The cardiovascular system is commonly used in psychophysiological research, partly because of its ability to indicate the state of the autonomic nervous system including stress levels (Berntson et al., 2017). Moreover, parameters like heart rate and blood pressure are straightforward to measure and quantify. However, the cardiovascular system is influenced by a complex array of factors, involving the autonomic and central nervous systems as well as the humoral (immune) system in the body. Consideration should be taken to aspects like participant’s age, activity level, changes in environment, and substances like caffeine, alcohol and tobacco (Jennings et al., 1981).

In SENSOLA, we have worked with heart rate and heart rate variability as cardiovascular indicators. Connections between heart rate and the sympathetic nervous system exist, but the findings are inconsistent (Valentini and Parati, 2009). For example, an increase in heart rate could be a sign of stress or anxiety (fight of flight), but also of cognitive load or increased physical activity. Heart rate has also been used as an indicator of stimuli involvement (Ulrich et al., 1991). Moreover, there are counterbalancing mechanisms, personal factors and spatiotemporal physiological patterns to take into account (Parsons and Tassinary, 2002; Berntson et al., 2017). Heart rate variability (HRV) concerns natural variations in heart rate and a higher HRV is generally associated with parasympathetic activity, particularly the high frequency component (Berntson et al., 1997).

To collect cardiovascular data, we have worked with both unobtrusive optical sensors (PPG) and electronic sensors (ECG), even though we have experienced limitations with PPG due to movement and light artefacts. For analysis with HRV, the exposure time is an important factor to consider in the experimental design with recommended standard windows of either five minutes or 24 h (Malik et al., 1996). Shorter windows can be used, but their reliability for HRV analysis is less certain (Pecchia et al., 2018).

#### 2.2.2 Electrodermal system

Electrodermal activity, EDA, concerns small changes in moisture as emitted by endocrine sweat glands in the skin (Dawson et al., 2016). As a biomarker, it has been used to assess everything from pain and attention to memory, emotions and decision making (Boucsein et al., 2012; Dawson et al., 2016). Yet, EDA is probably most known as an indicator of sympathetic nervous system arousal. However, due to its limitations in differentiating between positive and negative arousal, proper interpretation of skin conductance data requires triangulation with other physiological indicators and/or subjective data (Bradley and Lang, 2000; Figner and Murphy, 2011).

The term EDA is used interchangeably with skin conductance, eccrine activity and galvanic skin response. EDA can be subdivided in two types of indicators (Dawson et al., 2016); skin conductance level describes the continuous progression of conductance over time (tonic); skin conductance response, while still based on the skin conductance level, focuses on sudden responses which are highlighted by applying a frequency filter on the skin conductance level (phasic).

EDA can be measured on several locations on the body, including the soles of the feet, fingers (e.g., volar surfaces of distal phalange or medial phalange) and the palmar surfaces of the hand (thenar eminence and hyperthenar eminence) (Boucsein et al., 2012; Dawson et al., 2016). Because of its relation to skin moisture, factors like temperature and the participant’s level of physical activity should be considered in the study design (Wilhelm et al., 2006; Boucsein et al., 2012).

In SENSOLA, we opt to measure skin conductance with electrodes for best possible data quality. Depending on the study design, however, we also use less obtrusive devices like rings and wristbands. In our experience, data collected on the wrist is much less useful though, probably because the eccrine glands are less sensitive on the wrist. As indicators, we focus on mean levels of the tonic signal (SCL) as well as number of SCR/min and/or the area (integral) of the phasic signal.

#### 2.2.3 Respiratory system

Breathing is one of the most essential mechanical processes in the body, with several potential implications for psychophysiological research. Despite its apparent sensitivity to emotional states, cognition, and even odour perception (Lorig, 2017), respiration remains a relatively understudied area in psychological research compared to its prominence in medical fields. From a biophysical perspective, the purpose of breathing is to provide oxygen to the blood and also expel CO_2_ and other by-product gases produced by the body. The respiratory system thus involves ventilation (movement of air), diffusion (internal movement of gases) and perfusion (movement of blood in and out of organs) (Brashers, 2014). Breathing interacts with cardiovascular activity, such that the heart rate increase slightly on inspiration (Yasuma and Hayano, 2004), which is also an important contributing mechanism to heart rate variability. Breathing is typically quantified either by measuring how the torso moves or by assessing the gas exchange (such as breathing volume and blood oxygen levels) (Lorig, 2017).

In SENSOLA, we use two respiration transducers to register movement in the thoracic region and/or the abdominal region, focusing mainly on mechanical ventilation (respiratory effort) typically expressed as breaths per minute.

#### 2.2.4 Brain activity

Functional neuroimaging can be used to assess activity levels in different regions of the brain (Stern et al., 2001a; Geuter et al., 2016). Brain activity can be measured in different ways, including electronically [electroencephalography (EEG)], with a magnetic scanner [functional magnetic resonance (fMRI)] or by optical sensors [functional near infrared spectroscopy (fNirs)]. Application of EEG and fNirs is limited to the outer regions of the brain, while fMRI can scan the interior as well. On the other hand, fMRI has limited temporal resolution and is typically performed on stationary subjects lying in a tube, with severe implications for ecological validity.

SENSOLA uses fNirs and the setup includes a wearable cordless device focusing on the prefrontal cortex, as well as a more complex device capable of measuring multiple chosen regions of the brain simultaneously. This platform enables us to investigate the role of attention in human functioning within diverse real-world environments, building upon the well-established finding that nature can restore the capacity for directed attention (Kaplan and Kaplan, 1989; Berman et al., 2008).

#### 2.2.5 Eye movements and pupilometry

Vision is arguably one of the most important of the human senses and as such, eye tracking can be an invaluable source toward understanding human-environment interaction. Eye tracking data can reveal where, for how long, and in what order a subject has been focusing their vision during an experiment with obvious implications toward understanding visual attention (Carter and Luke, 2020). It should be noted that eye tracking tracks the focus of the gaze (not peripheral vision). Central parameters are fixations and saccades (saccades are movements between fixation points). The stimuli can be coded in terms of relevance as Areas Of Interests (AOI), which can be used to support the analysis process and identify potential correlation with other psychophysiological measurements.

Eye tracking data can also reveal information about the state of the pupil (pupilometry). Most notably, pupil diameter is an indicator that has been shown to correlate with cognitive load and arousal (Stern et al., 2001b). The behavior of the eyes can reveal the physiological state of a subject. For instance, some studies suggest that the notion of “mind wandering” is related to eye tracking parameters like divergence in eye movements, pupil dilation and eye blinking frequency (Smallwood and Schooler, 2015).

In SENSOLA, we use a mobile eye tracker with a software extension that allows it to function as both a screen-based device in controlled settings indoors, as well as in the field. It is possible to define AOI in both screen based stimuli and real world environments. However, due to the variable conditions of outdoor environments, certain indicators, such as pupil diameter, can be difficult to measure accurately, as global luminance significantly influences pupil size (Nguyen et al., 2022). This presents a particular challenge in outdoor settings, where controlling for fluctuating light levels is difficult.

### 2.3 Data about the environment

In people environment interaction studies, it is essential to be able to pinpoint in detail the environmental conditions that participants are exposed to over time. This data typically includes geographic location, environment type, environmental factors (e.g., noise levels, light conditions or temperature), and occurring events or social activities. Environmental data can be collected by dedicated devices, wearable sensors and/or extracted from databases. This data can then be synchronized with physiological measurements.

#### 2.3.1 Global Navigation Satellite System

A Global Navigation Satellite System (GNSS) uses satellite data to log geodata, which includes spatial coordinates and timestamps. Various GNSS systems exist, with the most common being the Global Positioning System (GPS). SENSOLA utilizes GNSS loggers that primarily rely on GPS data. However, we also employ more advanced units that can track data from multiple satellite systems simultaneously, achieving accuracy down to below 1 meter. This continuous recording of spatial data serves several purposes in our research. First, it allows us to reference psychophysiological data to specific environmental exposures. Second, it helps ensure participants have adhered to designated paths during studies. Finally, GNSS data can be used to estimate walking speed. It’s important to note that GNSS signals can be affected by factors like dense tree cover or urban environments. To ensure accurate synchronization with GNSS data, other devices need to be set to the precise timestamp provided by the GNSS system itself.

#### 2.3.2 Accelerometer

Accelerometers are commonly found in modern smartphones and other devices like pedometers. They measure acceleration, but not speed directly. This means the data does not necessarily indicate the distance traveled (an accelerometer in constant motion would not generate data). In SENSOLA, we have primarily used accelerometers for synchronization purposes. For instance, shaking a sensor containing an accelerometer while video filming creates a mutual event marker that can be used to align data streams during later processing. Additionally, many modern physiological data recording devices also include integrated accelerometers. Accelerometer data is valuable for providing an overview of participant behavior during an experiment. By analysing changes in acceleration patterns, we can distinguish between walking phases and stationary periods during an experiment.

#### 2.3.3 Still images

Still images serve various purposes in our research. They can be used for documentation, such as capturing the layout and key features of an experimental environment or demonstrating the setup of specific equipment. Additionally, photography can be integrated into the experimental design. For instance, participants might be prompted to document their surroundings during a study, focusing on specific features or capturing anything they find noteworthy. Still images can also be employed as controlled visual stimuli presented in an indoor setting.

#### 2.3.4 Audio-visual data

For a comprehensive understanding of psychophysiological responses in real-world settings, video with sound plays a central role in the SENSOLA setup. Audio-visual data allows us to document real-world experiments (*in situ*) and capture the environmental context, which is crucial for interpreting psychophysiological responses after the experiment. The video documentation provides a rich reference point for understanding the character of the environment, aiding interpretation of physiological data. Additionally, video helps us track significant events that occur during the experiment, allowing us to identify potential disturbances or particularly engaging experiences. Furthermore, video recorded outdoors can be used as a representation of the environment and played back as stimuli in the indoor laboratory, facilitating methodological comparisons between indoor and outdoor experiments. Video also serves as a tool to support interview sessions, where participants can reflect on their environmental experiences by watching footage of the setting they encountered in retrospect (Cerwén, 2024). Video elicitation in interviews is particularly valuable as it separates the environmental experience and physiological measurement from the evaluation phase, helping to keep the environmental experience as natural as possible. Additionally, video can assist participants in recalling and articulating their experiences more accurately.

SENSOLA utilizes various video recording devices, including action cameras, video glasses, and a mobile eye tracking camera. The video data is synchronized with physiological data, typically achieved by filming a sensor containing an accelerometer to create a common reference point during later processing.

#### 2.3.5 Sound

We’ve found that the sound quality from built-in microphones in many video cameras is often insufficient. If high-quality audio is of importance, an external field recorder is a better option with significant improvements in audio fidelity. Proper wind shielding is essential to minimize wind noise. When attaching a recorder to a participant, consideration should be given to the sound of footsteps, breathing, or clothing rubbing (e.g., in a pocket). Sounds from the participant can be regarded as noise, but these sounds can also be valuable. For instance, footsteps can help identify movement artefacts in physiological data, while breathing patterns, sighs, or laughter might provide insights into emotional responses. Sound is also essential for understanding activities happening around the participant, which might not be visible on video.

The lab has various audio recording devices, including stereo recorders and equipment for capturing sound environments in first-order ambisonics (four channels) for processing and binaural presentation with VR. Additionally, we use a Class 1 sound level meter to measure sound pressure levels, with the capability to log data over time and record a reference audio file.

#### 2.3.6 Weather

Weather is an important factor in outdoor experiments, as it has repercussions both on experience (Knez et al., 2009) and sometimes also on the quality of data (Boucsein et al., 2012; Przybyło et al., 2016). In some studies, researchers might try to control or minimize the influence of weather on the experiment (Gidlow et al., 2016), while in others, weather itself might be the topic of investigation (Smalley and White, 2023). Regardless of the research focus, considering general weather conditions is crucial during the experimental design phase.

Timestamps in collected data can serve as a reference for later retrieval of corresponding weather data from historical weather databases. Additionally, video recordings can provide clues about microclimatic variations, such as cloud cover affecting sunlight exposure or the participant walking under trees.

While SENSOLA has not yet employed specific methods for objectively recording outdoor weather data, depending on the research question, it might be relevant to measure specific factors like daylight conditions. Daylight is known to influence various aspects, including preference (Beute and de Kort, 2013) and mood (Schwarz and Clore, 1983). The light conditions in the indoor lab has been measured and calibrated. Some physiological measurement equipment in our lab also include the capability for thermal imaging to record body temperature.

### 2.4 Synchronization

Synchronization is critical for integrating various data streams in the SENSOLA lab, including psychological, physiological, and environmental data. We use a combination of automated and manual synchronization, depending on the study design and equipment employed. The main system automatically synchronizes physiological and accelerometer data. While manual methods like timestamps, event markers, and accelerometer data can be time-consuming and prone to errors, they are often necessary to complement data recorded with the main system.

For instance, to synchronize video with other data streams, we use an accelerometer. By briefly shaking a sensor containing an accelerometer while filming, a distinct movement is recorded in both the accelerometer data and the video, providing a common reference point for precise manual alignment during data processing.

Timestamps are a straightforward means of aligning data, provided the internal clocks of all devices are accurate. It’s important to note that offline devices need to be manually updated before an experiment starts, as clock drift can be substantial. Equipment with event marker buttons can be used for additional synchronization, but in our experience, focusing on accurate time stamping is more reliable.

## 3 SENSOLA setup and equipment

**Figure 2:**
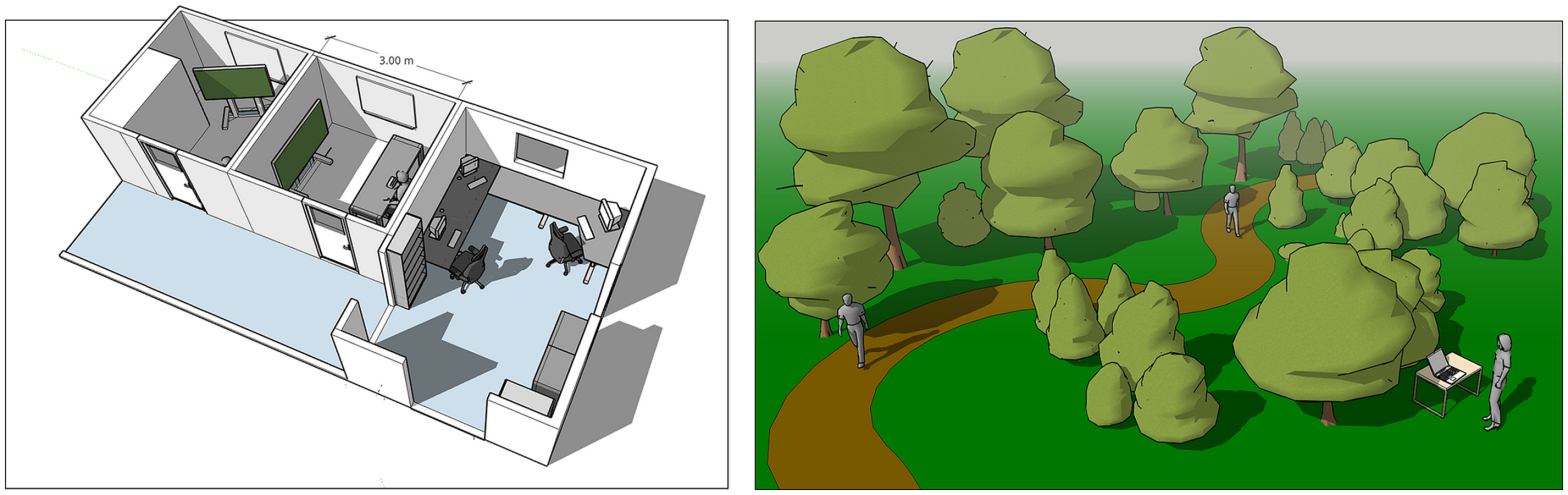
Left panel: A 3D sketch of the indoor laboratory in SENSOLA. Right panel: An illustration of a hypothetical experiment conducted on-site, using portable equipment from the SENSOLA laboratory.

SENSOLA consists of two sections, a traditional indoor facility and a portable set of equipment for outdoor studies (See Figure 2). Much of the equipment can be used in both indoor and outdoor settings. Both sections centres on the same main system for data handling.

### 3.1 Main system

**Figure 3:**
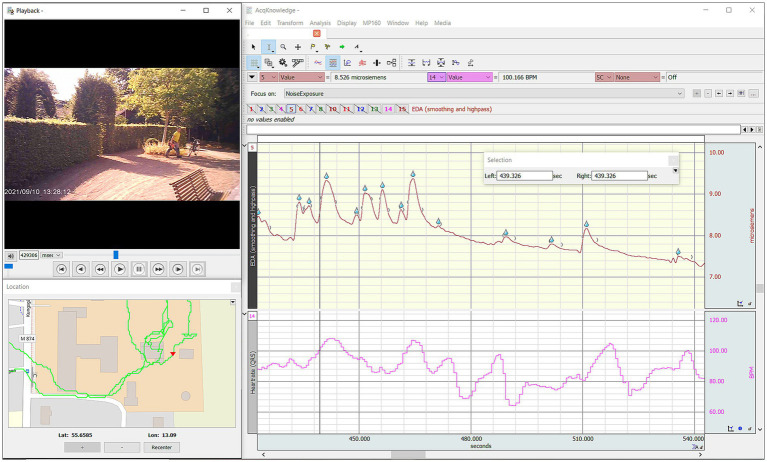
A screenshot of an Acknowledge session from SENSOLA pilot 2. Top left: Participant’s view (audiovisual data) from video glasses. Bottom left: GPS data. Right: Physiological data (skin conductance level and heart rate highlighted). During a noise intervention in this section, the participant’s skin conductance level increases, which may be indicative of heightened arousal.

At the core of the SENSOLA lab’s data handling is Biopac’s Bionomadix system and the associated Acqknowledge software (See Figure 3). This software allows for simultaneous visualization of multiple physiological data streams alongside video and GPS data. All physiological data recorded with the system is automatically synchronized. Time-series data from other systems can be imported via text files for manual synchronization. Data for each participant is kept separate and can be exported for further analysis in external statistical software.

### 3.2 Indoor facilities

The indoor facility houses a control room and two traditional experimental rooms for stimuli presentation (See Figure 2). The control room is separated from the experimental rooms to minimize disturbances (Curtin et al., 2007). The experimental rooms are equipped with large OLED screens for audio-visual representations. Each screen is connected to a dedicated computer situated in the control room, equipped with a Nvidia Quadro RTX 4000 graphics card. Stimuli are presented from the control room computers using E-prime 3.0 (Psychology Software Tools).

In experiment room 1, there is a Chronos response box (integrated with E-prime 3.0). Participants use the response box to interact with the experiment, for example by answering questions in a microphone or responding to questionnaires with Likert scales. A keyboard can also be connected for written responses.

Physiological responses are measured using the Biopac BioNomadix. This system consists of mobile amplifiers worn by the participant and a wireless receiver (MP160 + PPGED-R/RSPEC-R) located in the control room connected to a dedicated computer. Data is transferred wirelessly in real-time to the computer where the Acqknowledge 6.0 software is used for recording. The BioNomadix system uses wireless digital RF technology at 2.4 GHz (bi-directional) (Biopac, 2020). The current BioNomadix setup in SENSOLA facilitates measurement of ECG, Respiration, Skin conductance and Pulse Plethysmography (BN-RSPEC and BN-PPGED).

The setup also includes a pair of mobile eye tracking glasses (ETVision from Argus Science) with an addon for screen-based stimuli (StimTrac and ETAnalysis). The eye tracker can be integrated with Biopac for automatic synchronization or used independently.

To synchronize stimuli and measurements, we use trigger signals sent from E-Prime to the Biopac software AcqKnowledge (via the Chronos box equipped with a Biopac STP-C expansion and a DB25 parallel cable). As an additional synchronization method, one of the experimental rooms is equipped with a LED light (Biopac OUT103) that can be triggered from AcqKnowledge, and used as a synchronization event. Both experimental rooms are equipped with Logitech StreamCams with possibility to stream video directly to AcqKnowledge.

For data analysis, we use a combination of software, mainly Mircosoft Excel, Open Office Calc, R and Minitab. Kubios HRV Premium 3.5 is used for heart rate variability analysis.

### 3.3 Portable equipment

The portable setup utilizes the same BioNomadix amplifiers as the indoor system, but data is recorded using a portable logger instead of a computer connection. The logger has a wireless range of about one meter (Biopac, 2020) and should be kept close to the amplifiers (on the same side of the participant’s body). While the logger supports recording the same physiological measures as the indoor system, real-time data viewing is not available. However, a preview of the data stream can be viewed before recording starts.

For brain activity measurement, the lab employs fNirs technology from Artinis. Two devices are used: the Artinis Octamon^1^ designed for the prefrontal cortex and the Artinis Brite, a multi-channel device with flexible optodes for broader region coverage. Each device connects to a dedicated laptop via Bluetooth, and the Oxysoft Software controls data collection. Data can be imported into AcqKnowledge via txt or edf for manual synchronization based on timestamps.

In addition to the BioNomadix system, SENSOLA utilizes a range of smaller devices for specific situations. These include the Empatica E4^1^ wristband (measuring skin conductance, pulse plethysmography and temperature), the Polar H10 heart rate monitor (~130 Hz sampling rate) and the Moodmetric^1^/Nuanic ring (skin conductance). While some of these devices are consumer oriented products and offer convenience and accessibility, it’s important to consider potential limitations in data quality, sampling rate and access to raw data compared to research-grade equipment.

## 4 Four experimental typologies

This section outlines four experimental typologies, each representing a distinct level of control over the research environment. These typologies are based on experiences from the first ten studies carried out in the lab (See Supplementary Appendix 1). The typologies are used here to structure our understanding of various research approaches where physiology and environmental psychology is combined.

### 4.1 Controlled experiments in the lab

Experimental studies on human subjects in controlled indoor facilities have been used in several fields, including psychology, psychoacoustics, psychophysiology, linguistics, neuroscience and medicine. In environmental psychology, the laboratory setting has been employed for instance using image stimuli (Hung et al., 2023), image and sound (Carles et al., 1999), video (Ulrich et al., 1991), virtual reality (Annerstedt et al., 2013), and even multisensory approaches (Hedblom et al., 2019; Lyu et al., 2023).

The primary benefit of controlled laboratory environments is the ability to tightly control both the stimuli presented to participants and the measurements taken. Technological advancements have enabled increasingly immersive experiences, including interaction with simulated environments. However, the technology itself can introduce extraneous factors that need to be considered. A key challenge of indoor labs is the limitation in ecological validity. Assessing this limitation can be difficult, but comparative studies in both laboratory and real world settings may offer insights (c.f. Schöne et al., 2023). Combining findings from studies conducted across different settings through triangulation (c.f. Vigliocco et al., 2024) provides a more comprehensive understanding of a phenomenon.

### 4.2 Controlled experiments in the field

This category encompasses experiments conducted in real world settings, but with strict limitations on the variability of the environmental experience. This approach aims to retain some of the control offered by indoor labs while allowing access to natural multisensory stimuli. For instance, several studies have been carried out in research on forest bathing, where participants are asked to sit in selected spots in a forest while measurements are taken, which are subsequently compared with a reference environment (Stier-Jarmer et al., 2021). To control for external influences, the research area may be controlled for other visitors (Coss and Keller, 2022) and/or designed with specific interventions (South et al., 2015). Weather can be controlled to some extent by choosing to do experiments only under the same conditions (Gidlow et al., 2016).

Controlled experiments outdoors can help to assess certain types/aspects of an embodied and multisensory environmental experience, but the ecological validity can be questioned as (a) the participant’s freedom is severely limited, and (b) “disturbances” from the outside are controlled.

### 4.3 Semi-controlled experiments in the field

Semi-controlled experiments aim to mimic real-world conditions as closely as possible while maintaining some degree of experimental control. In these studies, participants engage in tasks within the chosen environment, and events occurring in the environment are considered part of the experiment. A predefined set of expected events can be identified and listed before the experiment (e.g., human interactions, encounters with vehicles), and other events may be excluded. By surveying events that naturally recur and their frequency, the setting can be described comprehensively, which helps determine when experiments should be conducted to match typical conditions for the given location. Examples of semi-controlled experiments include having participants walk along a designated trail, (c.f. Song et al., 2014), or following specific instructions such as “you should go through this forest, cross this landmark and then come back through this park.” Research designs might also involve specific activities, such as driving a car (Johnson et al., 2011).

Semi-controlled experiments offer a potential balance between ecological validity and control. However, they might be criticized for not fully achieving either. A major advantage compared to experiments in everyday life is the possibility to maintain control over equipment and data quality as the setting and timeframe is limited.

### 4.4 Experiments in everyday life

The least level of control will arguably be achieved when participants are free to go about their daily lives as usual, while data is recorded. Data obtained from participants’ own smart devices might be considered an option with potential logistic benefits. Participants can also be asked to wear a dedicated unobtrusive device with long-term measurement capabilities, such as a pulse belt or a smart wrist watch combined with GPS. While relying on participants to oversee data collection can be efficient, it can also make it challenging to assess internal validity (even if ecological validity is high) due to the potential for low data quality.

Examples of approaches in everyday life also include experience sampling and EMA (Intille, 2012; Kubiak and Krog, 2012), which have included, among other things, studies on health and wellbeing during work (Eatough et al., 2016) and leisure (Kono et al., 2022).

From a psychophysiological point of view, challenges with experiments in everyday life could be large datasets, lack of data control, confounding factors, and ethical considerations.

## 5 *In situ* guidelines for environmental psychophysiology

This section outlines general considerations for conducting psychophysiological studies in real-world settings (*in situ*). While the focus is on semi-controlled environments, these guidelines may also be applicable to other in situ contexts.

### 5.1 Sitting still or moving

If a participant is involved in a physical activity like walking, it is important to be aware that any changes in physiological responses could be due to the physical activity itself rather than external stimuli from the environment. This is further complicated by the fact that arousing environmental stimuli can influence walking pace (Franek, 2013), and that exercise, especially medium intensity, can lead to an increase in arousal (Kamijo et al., 2004). Furthermore, even during stationary measurements, prior physical activity may have lingering physiological effects, such as elevated heart rate or respiration. Physical activity is particularly influential for cardiorespiratory variables like heart rate and breathing rate (Rohrbaugh, 2016), but can also affect EDA and other measurements (Wilhelm et al., 2006). In addition to changes in physiological responses, there are also an increased risk for artefacts to consider when a participant is moving.

### 5.2 Study location/s

**Figure 4:**
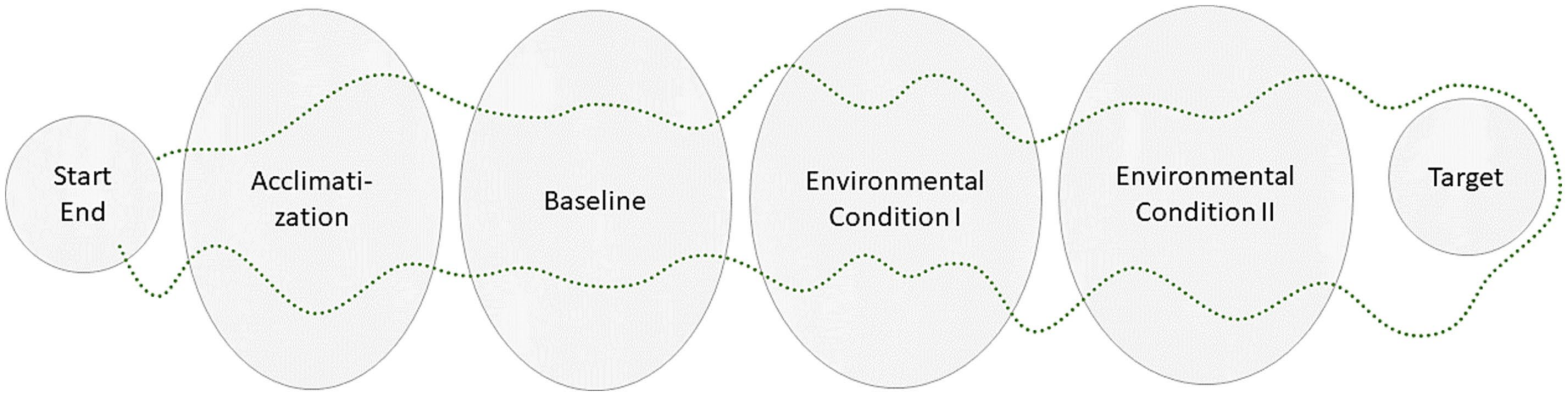
Hypothetical layout for an on-site walking experiment with start/end (base), acclimatization, baseline, and two environmental conditions. If a target destination is used (e.g., walk to a specific point and return), the participant will be exposed to both environmental conditions twice with reversed order.

A thorough examination of the chosen study location is crucial to ensure its suitability for the research. Key features like environmental types, transitions, events, paths, and overall layout should be mapped for reference. For logistical purposes, identifying a designated base (e.g., nature centre, athletics changing facility, or library) to welcome participants and house equipment is ideal. Planning should consider the starting location, baseline measurement area, walking paths, potential interventions, targets and environmental exposures (see Figure 4 for a hypothetical layout).

Field studies have limitations in randomizing stimuli compared to labs. However, reversing the walking path direction can address order effects to some extent. Additionally, exposing participants to the same stimuli more than once can offer statistical advantages. Potential on-site events should be considered and mapped for reference. If the area is open to the public, social encounters become an important factor to consider during design.

### 5.3 Participants and recruitment

Recruiting participants for outdoor experiments, especially in remote areas, presents challenges. Limited accessibility and increased time commitment can hinder finding a representative sample. Choosing locations accessible by public transportation or offering transportation assistance (Liu et al., 2021) can address accessibility concerns. For studies of national parks and other remote areas, participants have often been recruited on site among visitors (see, e.g., Levenhagen et al., 2021). This could be a viable strategy for unobtrusive wearable studies, but participant bias is a potential risk.

### 5.4 Managing participants and applying equipment

Mobile experiments typically require participants to carry equipment in accessory bags or with straps. To minimize signal obstruction, equipment needing to communicate should be positioned on the same side of the body.

Electrode application often starts due to their settling time. Mounting torso electrodes (ECG) can be perceived as intrusive, potentially raising stress levels due to physical contact and intrusion of private space (c.f. Gale and Smith, 1980). In SENSOLA, we are testing self-application with instructions and pre-experiment signal checks. This approach has yielded positive results.

It’s crucial to consider the physical and psychosocial environment participants encounter, and acknowledge the risk that participants could experience levels of stress or even anxiety during the onset of an experiment (Gale and Smith, 1980). Clear communication can help participants feel informed, safe, and calm about the experiment and equipment.

For ECG and skin conductance, it is important to check that chords for ECG and skin conductance do not interfere with the participant’s clothing. Even minor pulling can cause artefacts and data loss. Securing cords with surgical or sports tape can mitigate this risk (Biopac, 2024).

### 5.5 Baseline

Baseline measurements provide a reference point for comparing data collected during stimuli exposure (Stern et al., 2001c). A period of acclimatization allows participants to adjust to the experimental setting before baseline data collection. In laboratory settings, it has previously been found that cardiovascular activity can stabilize after about one minute (Gerin et al., 1994), and for skin conductance, a total baseline session of between 2 to 4 min have been suggested (Braithwaite et al., 2013). Unlike stationary settings, we suggest a slightly longer acclimatization for subjects in movement to allow for both bodily and mental adjustment. Electrode settling time is also a factor, typically requiring around five minutes (Braithwaite et al., 2013; Biopac, 2024). It is recommended that the acclimatization period and baseline reading be combined into a single session, with the analysis period extracted later, using the appropriate delay relative to the experiment start (Quintana et al., 2016).

The actual measurement duration required can vary depending on the physiological variable, with heart rate variability requiring more time than heart rate or skin conductance. Importantly, too long baseline sessions can trigger a response in some participants due to factors like restlessness, boredom, internal thoughts, emotional processing or anxiousness regarding the progression of the experiment (c.f. Stern et al., 2001c). For indoor studies, it has been suggested that non-demanding tasks during the baseline can be used to address such challenges (Jennings et al., 1992; Krebl et al., 2021). For studies *in situ*, problems with under stimulation during baseline should be less problematic.

The concept of a neutral baseline environment is crucial, but neutrality may need to be defined in context. Moreover, when participants are moving (e.g., walking), the baseline should account for the physiological changes caused by the movement itself. Terminology like “walking baseline” or “action baseline” can be used for clarity.

### 5.6 Unexpected events and confounders

Unlike controlled laboratory experiments that eliminate variations in environmental stimuli, real-world studies involve unexpected events that can disrupt the intended design. For instance, a noisy protest during a park study might invalidate data about the park environment itself. These disruptions are called confounding variables.

However, unexpected events (e.g., encountering people, animals, vehicles, work activities) are an intrinsic part of real-world experiences, and their presence enhances the study’s ecological validity, or the generalizability of findings to real-world settings. Nevertheless, they need to be addressed in the research design.

Site visits and analysis help identify events commonly occurring in a specific location. These expected events can be distinguished from true confounders that significantly deviate from the typical experience. Private areas tend to have fewer unexpected events compared to public spaces.

There are also strategies for addressing unexpected events themselves. Increasing exposure time or repeating exposures can help average out their effects, allowing for generalizations about the environment. Events can also be tagged during data analysis. This allows for comparing physiological responses during events with non-event periods. This can be used to either compensate for unexpected events or study them directly.

Environmental changes can also be deliberately introduced as stimuli. For instance, COVID-19 restrictions allowed for comparing soundscapes before and during the pandemic (Aletta et al., 2020).

## 6 Concluding remarks

This paper summarizes our experiences building a laboratory designed for real-world (*in situ*) psychophysiological measurements. We discussed the possibilities and challenges of ambulatory measurements, and suggested a guideline for future research projects. Real-world studies present specific challenges that require careful research design. This paper highlighted several pragmatic considerations, including confounding factors, data quality, procedures, and site analysis. Our experience suggests that, despite the many challenges, well-designed in situ studies can generate valuable data with significant potential.

For instance, in situ studies can enhance our understanding of the mechanisms underlying individual experiences in both natural and urban settings. Multisensory experiences are difficult to simulate in a laboratory setting, and even if perfectly simulated, the artificial environment likely influences participant responses. *In situ* studies help us explore contextual factors such as the role of unexpected events and their impact on participants, the effect of social proximity and encounters on experiences, the influence of time of day, season, and weather as experienced in real-world situations, and how transitions between environments affect experiences.

These questions involve complex, nuanced variables that require a multifaceted methodological approach. This may include triangulation, combining physiological responses, self-reports from participants, and environmental data.

The research could be designed with different epistemological emphases in regards to the data being collected. For instance, a study with multiple participants could aim to make generalizations about an environmental stimulus, where the impact of specific events are averaged out. Alternatively, a qualitative study with fewer participants could focus on uncovering the complex psycho-social aspects of the environmental experience, supported by physiological data. These approaches can also be combined.

SENSOLA is operational and adaptable to various experimental setups. Current physiological responses measured in SENSOLA include real-time ECG, PPG, fNIRS, EDA, respiration, and eye tracking. While saliva cortisol, EMG, and blood pressure have been considered, they have not yet been implemented. We plan to integrate virtual reality capabilities for further methodological assessment and data triangulation. Additionally, augmented reality could bridge in situ and lab contexts, potentially simulating and assessing the effects of future environmental developments.

Most SENSOLA studies have so far been small-scale pilots. The experiences drawn from the studies have been summarized in this paper to serve as a peer-reviewed knowledge base for future research. The paper outlined four study types: controlled experiments in the lab, controlled experiments in the field, semi-controlled experiments in the field, and experiments in everyday life. Our primary focus has been on semi-controlled experiments, where we have found a good balance between experimental control and participants’ freedom. The overarching aim is to improve ecological validity while still maintaining internal and external validity.

## Data Availability

The original contributions presented in the study are included in the article/Supplementary material, further inquiries can be directed to the corresponding author/s.

## Footnotes

^1^Device is currently discontinued.

## References

[ref1] Aletta F. Oberman T. Mitchell A. Tong H. Kang J. (2020). Assessing the changing urban sound environment during the COVID-19 lockdown period using short-term acoustic measurements. Noise Mapping 7, 123–134. doi: 10.1515/noise-2020-0011

[ref2] Anciaes P. (2023). Effects of the roadside visual environment on driver wellbeing and behaviour – a systematic review. Transp. Rev. 43, 571–598. doi: 10.1080/01441647.2022.2133189, PMID: 40101104

[ref3] Ancora L. A. Blanco-Mora D. A. Alves I. Bonifácio A. Morgado P. Miranda B. (2022). Cities and neuroscience research: a systematic literature review. Front. Psych. 13. doi: 10.3389/fpsyt.2022.983352, PMID: 36440407 PMC9684645

[ref4] Annerstedt M. Jonsson P. Wallergard M. Johansson G. Karlson B. Grahn P. . (2013). Inducing physiological stress recovery with sounds of nature in a virtual reality forest - results from a pilot study. Physiol. Behav. 118, 240–250. doi: 10.1016/j.physbeh.2013.05.023, PMID: 23688947

[ref5] Berman M. G. Jonides J. Kaplan S. (2008). The cognitive benefits of interacting with nature. Psychol. Sci. 19, 1207–1212. doi: 10.1111/j.1467-9280.2008.02225.x 19121124

[ref6] Berntson G. G. Bigger J. T. Jr. Eckberg D. L. Grossman P. Kaufmann P. G. Malik M. . (1997). Heart rate variability: origins, methods, and interpretive caveats. Psychophysiology 34, 623–648. doi: 10.1111/j.1469-8986.1997.tb02140.x, PMID: 9401419

[ref7] Berntson G. G. Quigley K. S. Norman G. J. Lozano D. L. (2017). “Cardiovascular psychophysiology” in Handbook of psychophysiology. 4th. eds. Cacioppo J. T. Tassinary L. G. Berntson G. G. (New York, NY, US: Cambridge University Press), 183–216.

[ref8] Berryman D. R. (2012). Augmented reality: a review. Med. Ref. Serv. Q. 31, 212–218. doi: 10.1080/02763869.2012.670604 22559183

[ref9] Beute F. de Kort Y. A. W. (2013). Let the sun shine! Measuring explicit and implicit preference for environments differing in naturalness, weather type and brightness. J. Environ. Psychol. 36, 162–178. doi: 10.1016/j.jenvp.2013.07.016, PMID: 40191147

[ref10] Beute F. de Kort Y. IJsselsteijn W. (2016). Restoration in its natural context: how ecological momentary assessment can advance restoration research. Int. J. Environ. Res. Public Health 13:420. doi: 10.3390/ijerph13040420, PMID: 27089352 PMC4847082

[ref12] Biopac (2020). BioNomadix logger user manual: Wearable data logging system for use with BioNomadix transmitters and AcqKnowledge software. Goleta: Biopac Systems Inc.

[ref11] Biopac (2024). Introductory ECG guide. Available at: https://www.biopac.com/wp-content/uploads/ECG-Guide.pdf (Accessed February 15, 2024).

[ref13] Boucsein W. Fowles D. C. Grimnes S. Ben-Shakhar G. Roth W. T. Dawson M. E. . (2012). Publication recommendations for electrodermal measurements. Psychophysiology 49, 1017–1034. doi: 10.1111/j.1469-8986.2012.01384.x, PMID: 22680988

[ref14] Bradley M. M. Lang P. J. (2000). “Measuring emotion: behavior, feeling, and physiology” in Cognitive neuroscience of emotion (New York, NY, US: Oxford University Press), 242–276.

[ref15] Brady E. Prior J. (2020). Environmental aesthetics: a synthetic review. People Nature 2, 254–266. doi: 10.1002/pan3.10089

[ref16] Braithwaite J. J. Watson D. P. Z. Jones R. O. Rowe M. A. (2013). “Guide for Analysing Electrodermal activity & skin conductance responses for psychological experiments” in CTIT technical reports series.

[ref17] Brashers V. L. (2014). “Structure and function of the pulmonary system” in Pathophysiology: The biologic basis for disease in adults and children. eds. McCance K. L. Huether S. E. (St. Louis, Missouri: Elsevier).

[ref18] Bratman G. N. Hamilton J. P. Daily G. C. (2012). The impacts of nature experience on human cognitive function and mental health. Ann. N. Y. Acad. Sci. 1249, 118–136. doi: 10.1111/j.1749-6632.2011.06400.x, PMID: 22320203

[ref19] Brown S. C. Lombard J. (2014). “Neighborhoods and social interaction” in Wellbeing and the environment. eds. Cooper R. Burton E. Cooper C. L. (Blackwell: John Wiley & Sons).

[ref20] Carles J. L. Barrio I. L. de Lucio J. V. (1999). Sound influence on landscape values. Landsc. Urban Plan. 43, 191–200. doi: 10.1016/s0169-2046(98)00112-1

[ref21] Carter B. T. Luke S. G. (2020). Best practices in eye tracking research. Int. J. Psychophysiol. 155, 49–62. doi: 10.1016/j.ijpsycho.2020.05.010, PMID: 32504653

[ref22] Castaneda D. Esparza A. Ghamari M. Soltanpur C. Nazeran H. (2018). A review on wearable photoplethysmography sensors and their potential future applications in health care. Int J Biosens Bioelectron 4, 195–202. doi: 10.15406/ijbsbe.2018.04.00125 30906922 PMC6426305

[ref23] Cerwén G. (2024). “Assessing psychophysiological responses to environmental stimuli in-situ. A pilot study using wearable sensors,” in Abstract retrieved from the 8th annual BrEPS conference.

[ref24] Coss R. G. Keller C. M. (2022). Transient decreases in blood pressure and heart rate with increased subjective level of relaxation while viewing water compared with adjacent ground. J. Environ. Psychol. 81:101794. doi: 10.1016/j.jenvp.2022.101794

[ref25] Costall A. (1995). Socializing affordances. Theory Psychol. 5, 467–481. doi: 10.1177/0959354395054001

[ref26] Curtin J. J. Lozano D. L. Allen J. J. B. (2007). “The psychophysiological laboratory” in Handbook of emotion elicitation and assessment. eds. Coan J. A. Allen J. J. B. (New York: Oxford University Press).

[ref27] Dawson M. E. Schell A. M. Filion D. L. (2016). “The Electrodermal system” in Handbook of psychophysiology. eds. Berntson G. G. Cacioppo J. T. Tassinary L. G.. 4th ed (Cambridge: Cambridge University Press), 217–243.

[ref28] Eatough E. Shockley K. Yu P. (2016). A review of ambulatory health data collection methods for employee experience sampling research. Appl. Psychol. Int. Rev. 65, 322–354. doi: 10.1111/apps.12068, PMID: 40189918

[ref29] Figner B. Murphy R. O. (2011). “Using skin conductance in judgment and decision making research” in A handbook of process tracing methods for decision research. eds. Schulte-Mecklenbeck M. Kuehberger A. Ranyard R. (New York: Psychology Press), 163–184.

[ref30] Franek M. (2013). Environmental factors influencing pedestrian walking speed. Percept. Mot. Skills 116, 992–1019. doi: 10.2466/06.50.pms.116.3.992-1019 24175468

[ref31] Gale A. Smith D. (1980). “On setting up a psychophysiological laboratory” in Techniques in psychophysiology. eds. Martin I. Venables P. H. (Chichester: Wiley).

[ref32] Gerin W. Pieper C. Pickering T. G. (1994). Anticipatory and residual effects of an active coping task on pre- and post-stress baselines. J. Psychosom. Res. 38, 139–149. doi: 10.1016/0022-3999(94)90087-6, PMID: 8189403

[ref33] Geuter S. Lindquist M. A. Wager T. D. (2016). “Fundamentals of functional neuroimaging” in Handbook of psychophysiology. eds. Berntson G. G. Cacioppo J. T. Tassinary L. G.. 4th ed (Cambridge: Cambridge University Press), 41–73.

[ref34] Gibson J. J. (1979). The ecological approach to visual perception. Boston, MA, US: Houghton, Mifflin and Company.

[ref35] Gidlow C. J. Jones M. V. Hurst G. Masterson D. Clark-Carter D. Tarvainen M. P. . (2016). Where to put your best foot forward: psycho-physiological responses to walking in natural and urban environments. J. Environ. Psychol. 45, 22–29. doi: 10.1016/j.jenvp.2015.11.003

[ref36] Gifford R. Steg L. Reser J. P. (2011). “Environmental psychology,” in IAAP handbook of applied psychology. ed. Martin P. R. (Chichester: Wiley-Blackwell), 440–470.

[ref37] Hagerhall C. M. Laike T. Taylor R. P. Küller M. Küller R. Martin T. P. (2008). Investigations of human EEG response to viewing fractal patterns. Perception 37, 1488–1494. doi: 10.1068/p5918, PMID: 19065853

[ref38] Haynes S. N. Yoshioka D. T. (2007). Clinical assessment applications of ambulatory biosensors. Psychol. Assess. 19, 44–57. doi: 10.1037/1040-3590.19.1.44, PMID: 17371122

[ref39] Hedblom M. Gunnarsson B. Iravani B. Knez I. Schaefer M. Thorsson P. . (2019). Reduction of physiological stress by urban green space in a multisensory virtual experiment. Sci. Rep. 9:10113. doi: 10.1038/s41598-019-46099-7, PMID: 31300656 PMC6625985

[ref40] Horiuchi M. Endo J. Takayama N. Murase K. Nishiyama N. Saito H. . (2014). Impact of viewing vs. not viewing a real Forest on physiological and psychological responses in the same setting. Int. J. Environ. Res. Public Health 11, 10883–10901. 25333924 10.3390/ijerph111010883 PMC4211012

[ref41] Houtveen J. H. de Geus E. J. C. (2009). Noninvasive psychophysiological ambulatory recordings: study design and data analysis strategies. Eur. Psychol. 14, 132–141. doi: 10.1027/1016-9040.14.2.132

[ref42] Hull R. B. IV Stewart W. P. (1992). Validity of photo-based scenic beauty judgments. J. Environ. Psychol. 12, 101–114. doi: 10.1016/S0272-4944(05)80063-5

[ref43] Hung S.-H. Pálsdóttir A. M. Ode Sang Å. Shahrad A. Liao H.-H. Hsu Y.-Y. . (2023). How restorative landscapes can benefit psychological and physiological responses: a pilot study of human–nature relationships in Sweden and Taiwan. Landsc. Res. 48, 1073–1090. doi: 10.1080/01426397.2023.2213634, PMID: 40101104

[ref44] Intille S. S. (2012). “Emerging technology for studying daily life,” in Handbook of research methods for studying daily life. eds. Mehl M. R. Conner T. S. (New York, NY, US: The Guilford Press), 267–282.

[ref45] Jennings J. R. Berg W. K. Hutcheson J. S. Obrist P. Porges S. Turpin G. (1981). Committee report. Publication guidelines for heart rate studies in man. Psychophysiology 18, 226–231. doi: 10.1111/j.1469-8986.1981.tb03023.x, PMID: 7291437

[ref46] Jennings J. R. Kamarck T. Stewart C. Eddy M. Johnson P. (1992). Alternate cardiovascular baseline assessment techniques: vanilla or resting baseline. Psychophysiology 29, 742–750. doi: 10.1111/j.1469-8986.1992.tb02052.x, PMID: 1461961

[ref47] Johnson M. J. Chahal T. Stinchcombe A. Mullen N. Weaver B. Bédard M. (2011). Physiological responses to simulated and on-road driving. Int. J. Psychophysiol. 81, 203–208. doi: 10.1016/j.ijpsycho.2011.06.012, PMID: 21726587

[ref001] Kamijo K. Nishihira Y. Hatta A. Kaneda T. Kida T. Higashiura T. . (2004). Changes in arousal level by differential exercise intensity. Clinical Neurophysiology: Official Journal of the International Federation of Clinical Neurophysiology 115, 2693–2698. doi: 10.1016/j.clinph.2004.06.016 15546777

[ref48] Kaplan R. Kaplan S. (1989). The experience of nature: A psychological perspective. Cambridge: Cambridge University Press.

[ref49] Knez I. Thorsson S. Eliasson I. Lindberg F. (2009). Psychological mechanisms in outdoor place and weather assessment: towards a conceptual model. Int. J. Biometeorol. 53, 101–111. doi: 10.1007/s00484-008-0194-z, PMID: 19034531

[ref50] Kono S. Ito E. Gui J. J. (2022). Leisure's relationships with hedonic and Eudaimonic well-being in Daily life: An experience sampling approach. Leis. Sci. 47, 391–410. doi: 10.1080/01490400.2022.2102097, PMID: 40101104

[ref51] Korpilo S. Nyberg E. Vierikko K. Ojala A. Kaseva J. Lehtimäki J. . (2024). Landscape and soundscape quality promote stress recovery in nearby urban nature: a multisensory field experiment. Urban For. Urban Green. 95:128286. doi: 10.1016/j.ufug.2024.128286, PMID: 40191147

[ref52] Krebl M. Podlesek A. Geršak G. (2021). “A study of baseline in psychophysiological experiments” in 8th European medical and biological engineering conference. eds. Jarm T. Cvetkoska A. Mahnič-Kalamiza S. Miklavcic D. (Springer International Publishing), 45–50.

[ref53] Kubiak T. Krog K. (2012). “Computerized sampling of experiences and behavior” in Handbook of research methods for studying daily life (New York, NY, US: The Guilford Press), 124–143.

[ref54] Levenhagen M. J. Miller Z. D. Petrelli A. R. Ferguson L. A. Shr Y.-H. Gomes D. G. E. . (2021). Ecosystem services enhanced through soundscape management link people and wildlife. People Nature 3, 176–189. doi: 10.1002/pan3.10156

[ref55] Liu Q. Wang X. Liu J. Zhang G. An C. Liu Y. . (2021). The relationship between the restorative perception of the environment and the physiological and psychological effects of different types of forests on university students. Int. J. Environ. Res. Public Health 18. doi: 10.3390/ijerph182212224, PMID: 34831980 PMC8620764

[ref56] Lorig T. S. (2017). “The respiratory system” in Handbook of psychophysiology. 4th ed (New York, NY, US: Cambridge University Press), 244–257.

[ref57] Lyu K. Brambilla A. Globa A. de Dear R. (2023). An immersive multisensory virtual reality approach to the study of human-built environment interactions. Autom. Constr. 150:104836. doi: 10.1016/j.autcon.2023.104836, PMID: 37519946 PMC10371809

[ref58] Malik M. Bigger J. T. Camm A. J. Kleiger R. E. Malliani A. . (1996). Heart rate variability: standards of measurement, physiological interpretation, and clinical use. Eur. Heart J. 17, 354–381. doi: 10.1093/oxfordjournals.eurheartj.a014868, PMID: 8737210

[ref59] Marquart H. Stark K. Jarass J. (2022). How are air pollution and noise perceived en route? Investigating cyclists’ and pedestrians’ personal exposure, wellbeing and practices during commute. J. Transp. Health 24:101325. doi: 10.1016/j.jth.2021.101325, PMID: 40191147

[ref60] McCunn L. J. (2024). “The rise and future of environmental neuroscience in environmental psychology” in Environmental Neuroscience. ed. Kühn S. (Cham: Springer Nature Switzerland), 19–27.

[ref61] Nassauer J. I. (1983). Framing the landscape in photographic simulation. J. Environ. Manag. 17, 1–16.

[ref62] Nguyen K. T. Liang W.-K. Juan C.-H. Wang C.-A. (2022). Time-frequency analysis of pupil size modulated by global luminance, arousal, and saccade preparation signals using Hilbert-Huang transform. Int. J. Psychophysiol. 176, 89–99. doi: 10.1016/j.ijpsycho.2022.03.011, PMID: 35367510

[ref63] Parsons T. D. (2015). Virtual reality for enhanced ecological validity and experimental control in the clinical, affective and social neurosciences. Front. Hum. Neurosci. 9. doi: 10.3389/fnhum.2015.00660, PMID: 26696869 PMC4675850

[ref64] Parsons R. Tassinary L. G. (2002). “Environmental psychophysiology” in Handbook of environmental psychology (Hoboken, NJ, US: John Wiley & Sons, Inc.), 172–190.

[ref65] Parsons R. Tassinary L. G. Ulrich R. S. Hebl M. R. Grossman-Alexander M. (1998). The view from the road: implications for stress recovery and immunization. J. Environ. Psychol. 18, 113–140. doi: 10.1006/jevp.1998.0086, PMID: 39885891

[ref66] Peake J. M. Kerr G. Sullivan J. P. (2018). A critical review of consumer wearables, Mobile applications, and equipment for providing biofeedback, monitoring stress, and sleep in physically active populations. Front. Physiol. 9. doi: 10.3389/fphys.2018.00743, PMID: 30002629 PMC6031746

[ref67] Pecchia L. Castaldo R. Montesinos L. Melillo P. (2018). Are ultra-short heart rate variability features good surrogates of short-term ones? State-of-the-art review and recommendations. Healthc Technol Lett 5, 94–100. doi: 10.1049/htl.2017.0090, PMID: 29922478 PMC5998753

[ref68] Przybyło J. Kańtoch E. Jabłoński M. Augustyniak P. (2016). Distant measurement of Plethysmographic signal in various lighting conditions using configurable frame-rate camera. Metrol. Measure. Syst. 23, 579–592. doi: 10.1515/mms-2016-0052

[ref69] Quintana D. S. Alvares G. A. Heathers J. A. J. (2016). Guidelines for reporting articles on psychiatry and heart rate variability (GRAPH): recommendations to advance research communication. Transl. Psychiatry 6:e803. doi: 10.1038/tp.2016.73, PMID: 27163204 PMC5070064

[ref70] Rao M. Prasad S. Adshead F. Tissera H. (2007). The built environment and health. Lancet 370, 1111–1113. doi: 10.1016/S0140-6736(07)61260-4 17868821

[ref71] Rohrbaugh J. W. (2016). “Ambulatory and non-contact recording methods” in Handbook of psychophysiology. eds. Berntson G. G. Cacioppo J. T. Tassinary L. G.. 4th ed (Cambridge: Cambridge University Press), 300–338.

[ref72] Schöne B. Kisker J. Lange L. Gruber T. Sylvester S. Osinsky R. (2023). The reality of virtual reality. Front. Psychol. 14. doi: 10.3389/fpsyg.2023.1093014, PMID: 36874824 PMC9975753

[ref73] Schwarz N. Clore G. L. (1983). Mood, misattribution, and judgments of well-being: informative and directive functions of affective states. J. Pers. Soc. Psychol. 45, 513–523. doi: 10.1037/0022-3514.45.3.513

[ref74] Shcherbina A. Mattsson C. M. Waggott D. Salisbury H. Christle J. W. Hastie T. . (2017). Accuracy in wrist-worn, sensor-based measurements of heart rate and energy expenditure in a diverse cohort. J Pers Med 7. doi: 10.3390/jpm7020003, PMID: 28538708 PMC5491979

[ref75] Shuttleworth S. (1980). The use of photographs as an environment presentation medium in landscape studies. J. Environ. Manag. 11, 61–76.

[ref76] Smalley A. J. White M. P. (2023). Beyond blue-sky thinking: diurnal patterns and ephemeral meteorological phenomena impact appraisals of beauty, awe, and value in urban and natural landscapes. J. Environ. Psychol. 86:101955. doi: 10.1016/j.jenvp.2023.101955, PMID: 40191147

[ref77] Smallwood J. Schooler J. W. (2015). The science of mind wandering: empirically navigating the stream of consciousness. Annu. Rev. Psychol. 66, 487–518. doi: 10.1146/annurev-psych-010814-015331, PMID: 25293689

[ref78] Song C. Ikei H. Igarashi M. Miwa M. Takagaki M. Miyazaki Y. (2014). Physiological and psychological responses of young males during spring-time walks in urban parks. J. Physiol. Anthropol. 33:8. doi: 10.1186/1880-6805-33-8, PMID: 24887352 PMC4041337

[ref79] South E. C. Kondo M. C. Cheney R. A. Branas C. C. (2015). Neighborhood blight, stress, and health: a walking trial of urban greening and ambulatory heart rate. Am. J. Public Health 105, 909–913. doi: 10.2105/ajph.2014.302526, PMID: 25790382 PMC4386540

[ref80] Steg L. van den Berg A.E. de Groot J.I.M. (2019). "Environmental psychology: history, scope and methods," in Environmental psychology: An introduction, eds. Steg L. Groot J.I.M. De. Hoboken, NJ: Wiley.

[ref81] Steg L. Vlek C. (2009). Encouraging pro-environmental behaviour: An integrative review and research agenda. J. Environ. Psychol. 29, 309–317. doi: 10.1016/j.jenvp.2008.10.004

[ref82] Stern R. M. Ray W. J. Quigley K. S. (2001a). “Brain: electroencephalography and imaging” in Psychophysiological recording (New York: Oxford University Press), 79–105.

[ref83] Stern R. M. Ray W. J. Quigley K. S. (2001b). “Eyes: Pupillography and electrooculography” in Psychophysiological recording (New York: Oxford University Press), 125–141.

[ref84] Stern R. M. Ray W. J. Quigley K. S. (2001c). Psychophysiological recording. New York: Oxford University Press.

[ref85] Stern R. M. Ray W. J. Quigley K. S. (2001d). “Psychophysiology” in Psychophysiological recording (New York: Oxford University Press), 3–11.

[ref86] Stier-Jarmer M. Throner V. Kirschneck M. Immich G. Frisch D. Schuh A. (2021). The psychological and physical effects of forests on human health: a systematic review of systematic reviews and Meta-analyses. Int. J. Environ. Res. Public Health 18:1770. 33670337 10.3390/ijerph18041770 PMC7918603

[ref87] Ulrich R. S. (1983). “Aesthetic and affective response to natural environment” in Human behavior and environment: Advances in theory and research. Vol. 6, behavior and the natural environment. eds. Altman I. Wohlwill J. F. (New York: Plenum Press).

[ref88] Ulrich R. S. Simons R. F. Losito B. D. Fiorito E. Miles M. A. Zelson M. (1991). Stress recovery during exposure to natural and urban environments. J. Environ. Psychol. 11, 201–230. doi: 10.1016/S0272-4944(05)80184-7

[ref89] Uttley J. Simpson J. Qasem H. (2018). “Eye-tracking in the real world: insights about the urban environment” in Handbook of research on perception-driven approaches to urban assessment and design. eds. Aletta F. Xiao J. (Hershey, PA, USA: IGI Global), 368–396.

[ref90] Valentini M. Parati G. (2009). Variables influencing heart rate. Prog. Cardiovasc. Dis. 52, 11–19. doi: 10.1016/j.pcad.2009.05.004, PMID: 19615488

[ref91] Vigliocco G. Conventino L. De Felice S. Gregorians L. Kewenig V. Mueller M. . (2024). Ecological brain: reframing the study of human behaviour and cognition. R. Soc. Open Sci. 11:240762. doi: 10.1098/rsos.240762 39525361 PMC11544371

[ref92] Weber A. M. Trojan J. (2018). The restorative value of the urban environment: a systematic review of the existing literature. Environmental Health Insights 12. doi: 10.1177/1178630218812805, PMID: 30505146 PMC6256310

[ref93] White M. P. Hartig T. Martin L. Pahl S. van den Berg A. E. Wells N. M. . (2023). Nature-based biopsychosocial resilience: An integrative theoretical framework for research on nature and health. Environ. Int. 181:108234. doi: 10.1016/j.envint.2023.108234, PMID: 37832260

[ref94] WHO (2003). “Healthy urban planning in practice: experience of European cities” in Report of the WHO City action group on healthy urban planning. eds. Barton H. Mitcham C. Tsourou C. (Copenhagen: WHO Regional Office for Europe).

[ref95] Wilhelm P. Perrez M. Pawlik K. (2012). “Conducting research in daily life: a historical review” in Handbook of research methods for studying daily life (New York, NY, US: The Guilford Press), 62–86.

[ref96] Wilhelm F. H. Pfaltz M. C. Grossman P. Roth W. T. (2006). Distinguishing emotional from physical activation in ambulatory psychophysiological monitoring. Biomed. Sci. Instrum. 42, 458–463, PMID: 16817651

[ref97] Yao W. Zhang X. Gong Q. (2021). The effect of exposure to the natural environment on stress reduction: a meta-analysis. Urban For. Urban Green. 57:126932. doi: 10.1016/j.ufug.2020.126932, PMID: 40191147

[ref98] Yasuma F. Hayano J. (2004). Respiratory sinus arrhythmia: why does the heartbeat synchronize with respiratory rhythm? Chest 125, 683–690. doi: 10.1378/chest.125.2.683, PMID: 14769752

[ref99] Zube E. H. Simcox D. E. Law C. S. (1987). Perceptual landscape simulations: history and Prospect. Landsc. J. 6, 62–80. doi: 10.3368/lj.6.1.62

