# Supplementary material for: Psychophysiological research in real-world environments: methodological perspectives from the SLU Multisensory Outdoor Laboratory
Source: Front Psychol. 2025 May 30;16:1432180. doi: 10.3389/fpsyg.2025.1432180 (PMC12162570; doi:10.3389/fpsyg.2025.1432180)
Supplement: Supplementary file 1 [file Supplementary_file_1.pdf]

frontiers

Appendix 1

1 An overview of studies carried out at SENSOLA

**Name of study:**	**Participants:**	**Data collection:**	**Equipment used and type of data*:**	**Aim:**	**Approach:**	**Data analysis:**	**Typology:**	**References:**
SENSOLA pilot 1	5	July 2021	• BioNomadix (EDA, PPG, ECG, RSP, Acc)<br>• Polar H10 (ECG)<br>• Empatica E4 (BVP, EDA, Temp, Acc)<br>• GoPro7<br>• Columbus v-990 GPS	To assess possibilities to measure psychophysiological responses in situ with different devices and technologies.	Baseline and stress stimuli (angry faces). Recovery with four different conditions: walking/sitting indoors and outdoors.	Descriptive statistics, comparing physiological response across time windows.	Controlled and Semi-controlled experiment in the field.	Conference proceedings: (Hägerhäll & Cerwén, 2022)
SENSOLA Pilot 2	8	September/ October 2021 and September 2023	• BioNomadix (EDA, PPG, ECG, RSP, Acc)<br>• Videoglasses, GoPro7 and Sony RX-100 II<br>• Columbus v-990 GPS and Columbus P-10 Pro<br>• Speakers: Ion Job Rocker and HK Go + Play Wireless<br>• Svantek SV971A SPL Meter	To develop and test a research protocol for assessing environmental experiences in situ with particular focus on stress and stress recovery.	Two methodological protocols were tested. 1. Participants walked along a predetermined path in a delimited garden with speaker interventions. 2) Walked freely to follow a goal in the University campus without interventions.	Within-subject design, comparing physiological and psychologicalresponses across time windows using Anova.	Semi-controlled experiment in the field.	Conference proceedings: (Cerwén, 2024)

Cerwén G and Hägerhäll CM (2025) Psychophysiological research in real-world environments: methodological perspectives from the SLU Multisensory Outdoor Laboratory. *Front. Psychol.* 16:1432180. doi: 10.3389/fpsyg.2025.1432180

SENSOLA Pilot 3	4	September/ October 2021	• BioNomadix (EDA, PPG) and BIOPAC MP160<br>• GoPro7	To evaluate the possibilities of assessing arousal connected with exposure to the smell of banana fly pheromones	Participants are exposed to different smells delivered via test tubes.	Visual assessment, comparing video documentation and physiological response.	Controlled experiment in the lab.	
Forest break	16	September-November 2021	• Imotions Mobile App (Beta) with proximity sensors<br>• Polar H10 (R-R Interval)<br>• Empatica E4 (BVP, EDA, Temp, Acc)<br>• Samsung Galaxy Note 9	To develop and test a protocol for measurement of stress during a workday, and during breaks in a specially designed forest room.	Participants wear equipment to measure stress levels during their workday. Two times a day they are triggered to take breaks in either a control room or a forest room.	Within-subject design, comparing physiological and psychological responses across time windows using Anova.	Experiment in everyday life.	Media coverage (Helsingborg , 2021; SLU, 2024)
SENSOLA Pilot 4	3	October-November 2022	• Artinis Octamon 8-channel fNirs<br>• BioNomadix (EDA, PPG, ECG, RSP, Acc)<br>• GoPro7 and Sony RX-100 II	Explore methods to assess attention restoration and stress reduction in situ.	Participants were asked to sit down while being exposed to 1) a baseline period 2) backward calculation task 3) recovery phase. The experiment was performed in a natural condition and a semi-urban condition.	Comparing physiological responses across time windows.	Controlled experiment in the field.	Conference proceedings (Stoltz, Stålhammar, & Cerwén, 2022)
Perception of Greenroofs	22	November 2022 – September 2023	• BioNomadix (EDA, PPG) and BIOPAC MP160<br>• Argus ETVision mobile eyetracker and Stim Trac<br>• LG OLED 77" TV	To study psychophysiolo gical effects of exposure to different green roofs	Participants were exposed to a series of 21 images of various green roofs on a large OLED screen, while eye tracking, pulse and skin conductance was recorded. Additional self-reports were collected afterwards in an analogue questionnaire.	Within-subject design, comparing physiological and psychological responses across 7s image exposures using Anova.	Controlled experiment in the lab.	

SENSOLA Pilot 5	2	March 2023	• Empatica E4 (BVP, EDA, Temp, Acc)<br>• BioNomadix (EDA, PPG, ECG, RSP, Acc)<br>• GoPro7<br>• Polar H10 (Kubios Mobile, R-R Interval)	To test the possibilities to measure physiological effects of relaxation exercises on horseback	Participants were taking part in an established therapy program while wearing devices to measure physiological responses.	Visual assessment, comparing video documentation and physiological response.	Controlled experiment in the field.	Media coverage (CEPI, 2022)
Erasmus student internship	7	May 2023	• Moodmetric ring (M-M)<br>• Moodmetric app<br>• Columbus P10 Pro	To test a consumer oriented device and measure potential stress reduction capabilities in four different kinds of green areas.	Participants wore equipment for 4-5 working days. Once a day, they were asked to take a break, walking in one of four pre-assigned areas. All data was synchronized and illustrated in GIS.	Descriptive statistics combining GIS and skin conductance data.	Semi-controlled experiment in the field.	Internship report (Bascoul, 2023)
SENSOLA Pilot 6	1	June – August 2023	• Moodmetric ring (M-M)<br>• Moodmetric app<br>• Columbus P10 Pro	To evaluate if skin conductance response show any consistent patterns across repeated walks, which could be attributed to the physical environment.	A single case study of 25 repetitions of the same recreational walk, comprising segments with clearly different outdoor characteristics and naturally occurring encounters with traffic, people and wildlife.	Descriptive statistics combining GIS and skin conductance data.	Semi-controlled experiment in the field.	Conference proceedings (Hägerhäll, 2024)

Master project	13	May 2024	• Videoglasses, GoPro7	To develop a new methodology to examine mind wandering in outdoor environments. To use the methodology to explore mind wandering in an urban blue-green space.	Participants walked along a predetermined path while video was recorded. Afterwards, they were asked to look at the video and note all instances of mind wandering.	Descriptive statistics, qualitative descriptions of mind wandering episodes.	Semi-controlled experiment in the field.	Master thesis (Staudinger, 2024)

*Type of data abbreviations:

Acc – Accelerometer

BVP – Blood volume pulse (Pulse measure based on PPG)

ECG – Electrocardiography (electric activity of the heart).

EDA – Electrodermal activity (skin conductance).

fNirs – Functional Near Infrared Spectroscopy (brain activity)

M-M Moodmetric level (Electrodermal activity, algorithm based on a combination of tonic and phasic responses)

PPG – Photoplethysmography (optical pulse sensor)

R-R Interval – Time difference between consecutive heart beats (for pulse and heart rate variability)

RSP – Respiration (breathing rate)

References:

Bascoul, L. (2023). *The impact of the environment on human health and behaviors : Two case studies on the human-nature relationship*. Retrieved from SLU Alnarp: https://res.slu.se/id/publ/126310

CEPI. (2022). Kan hästunderstödd terapi hjälpa vid stress och ångest? Retrieved from https://www.cepi.lu.se/artikel/kan-hastunderstodd-terapi-hjalpa-vid-stress-och-angest

Cerwén, G. (2024, July 11-12). *Assessing psychophysiological responses to environmental stimuli in-situ. A pilot study using wearable sensors.* Paper presented at the 8th annual BrEPS conference, Portsmouth, UK.

Helsingborg, S. (2021). Skogspaus – ett virtuellt skogsbad i vardagen. Retrieved from https://innovation.helsingborg.se/initiativ/skogspaus-ett-virtuellt-skogsbad-i-vardagen/

Hägerhäll, C. (2024, 2-5 July). *The everyday recreational walk: Assessed with a wearable sensor ring.* Paper presented at the 28th IAPS International Association People Environment Studies, Barcelona, Spain.

Hägerhäll, C., & Cerwén, G. (2022, July 5-8). *Taking the psychophysiology lab outdoors.* Paper presented at the IAPS Online Conference. IAPS 27 Conference Global Challenges – Local Impacts, Lisbon, Portugal.

SLU. (2024). Gröna multisensoriska inomhusmiljöer för ökat välbefinnande. Retrieved from https://www.slu.se/centrumbildningar-och-projekt/slu-future-one-health/forskning/OHprojekt/dnh/grona/

Staudinger, C. (2024). *Out for a Walk Where the Thoughts Take Flight : naturalistic experience sampling of mind wandering in an urban blue-green space.* (Master). Swedish University of Agricultural Sciences, Alnarp. Retrieved from https://stud.epsilon.slu.se/20653/

Stoltz, J., Stålhammar, S., & Cerwén, G. (2022, 8-10 Nov). *Exploring Neurophysiological Indicators of Human-Nature Relations: Potential Measures for Attention Restoration, Stress Reduction, and Nature Connectedness.* Paper presented at the Urban Transitions 2022: Integrating urban and transport planning, environment and health for healthier urban living, Sitges, Spain.
