# Supplementary material for: Psychophysiological research in real-world environments: methodological perspectives from the SLU Multisensory Outdoor Laboratory
Source: Front Psychol. 2025 May 30;16:1432180. doi: 10.3389/fpsyg.2025.1432180 (PMC12162570; doi:10.3389/fpsyg.2025.1432180)
Supplement: Supplementary file 2 [file Supplementary_file_2.pdf]

# *Appendix 2*

## 1 Data quality and treatment considerations

Field-based psychophysiological measurements are more susceptible to artefacts in the recording equipment due to factors such as movement, sunlight interference, and signal disruption in wireless protocols. While using appropriate equipment and deployment methods can mitigate many of these issues, some level of data cleaning and filtering is typically necessary. This appendix provides a general overview of commonly encountered artefacts and recommended data treatment procedures for measurements on: cardiovascular activity, electrodermal activity, respiration, brain activity, and eye movements and pupilometry.

### 1.1.1 Cardiovascular activity

ECG measurements using torso-mounted electrodes have shown good data quality during walking in moderate temperatures (20-25°C). However, sweat production in warmer climates or during exercise might necessitate special electrodes (Xie et al., 2022).

Outdoor environments can introduce occasional data loss due to movement (pulling of ECG chords) or signal interference. We address this by either manually inspecting QRS peaks in AcqKnowledge for accuracy and ectopic beats, or by utilizing Kubios HRV Premium, which offers missing data correction algorithms. We employ a range of HRV indices in both time (e.g. RMSSD) and frequency domains (e.g. HF $ms^2$).

For heart rate variability, it is important to consider the time window, as the length itself has accumulative implications on the result (Task Force et al., 1996). Suggested standards include long-term (24h) and short-term measurements (5min). Depending on the analysis, ultra short-term windows down to 30 seconds or even 10 seconds have been employed, even though the reliability is as yet unclear (Pecchia, Castaldo, Montesinos, & Melillo, 2018).

Notably, the recommended frequency band for respiratory sinus arrhythmia analysis (0,15Hz-0,4Hz) might require adjustment based on breathing patterns observed while participants perform activities (Berntson, Quigley, Norman, & Lozano, 2017). The default upper limit of 0,4Hz corresponds to 24 breaths per minute, but with exercise this can be adjusted up to 1 Hz (60 breaths per minute). Similarly, the lower limit of 0,15Hz corresponds to 9 breaths per minute. An adjustment upwards may have implications for the minimum time window required for analysis; while the standard 0,15Hz has a theoretical time window limit of about a minute (67s), an adjustment up to 0,3Hz (18 breaths per minute) would have a theoretical limit of only 30s) (c.f. Task Force et al., 1996). For children and infants, it is recommended to use a frequency band of 0,24-1,04 (Quintana, Alvares, & Heathers, 2016), which corresponds to a breathing rate between about 14 and 62 BPM.

Cerwén G and Hägerhäll CM (2025) Psychophysiological research in real-world environments: methodological perspectives from the SLU Multisensory Outdoor Laboratory. *Front. Psychol.* 16:1432180. doi: 10.3389/fpsyg.2025.1432180

### 1.1.2 Electrodermal activity

While sweat glands in the palms and feet are primarily triggered by emotions (Rohrbaugh, 2016) some studies suggest that high temperatures can have an influence as well (Boucsein et al., 2012). This is important to consider in outdoor environments where temperature fluctuations might occur.

The BIOPAC system typically captures EDA data at a high sampling frequency (1000-2000Hz). However, for analysis purposes, this data is typically downsampled to a more manageable frequency like 250Hz or 500Hz to reduce computational workload.

Outdoor recordings using BIOPAC BioNomadix may encounter occasional signal artifacts due to movement or interference. Fortunately, EDA is a relatively slow-moving physiological response. This allows us to effectively filter these artefacts using a technique called median smoothing with a window of 0.25 seconds (assuming a 500Hz sampling rate) (c.f. Braithwaite, Watson, Jones, & Rowe, 2013). Additionally, a 1Hz low-pass filter can be applied for further noise reduction.

EDA can be separated into two main components: tonic and phasic. The tonic component, reflected by Skin Conductance Level (SCL), represents continuous activity and is calculated as the average value over a specific time window (e.g., 2 minutes). Skin Conductance Responses (SCRs) should be excluded for this calculation (Boucsein et al., 2012).

The phasic component of EDA on the other hand, represent sudden increases in conductivity, also known as skin conductance responses (SCR). SCR is representative of a sudden rise in conductivity, which may or may not be related to an external stimuli. In case of stimuli-elicited SCR, there is a time window of about 1-4s between stimuli and the onset of the response (Boucsein et al., 2012).

SCR analysis involves calculating the number of SCRs per minute (SCR/Minute) and their peak levels. Typical values are between 1 and 3 SCR/minute for subjects at rest (Dawson, Schell, & Filion, 2016). A threshold between 0.01 and 0.05 microsiemens is commonly used to identify SCRs. However, in outdoor settings where movement artifacts are more likely, a slightly higher threshold of 0.04-0.05 microsiemens is recommended (Braithwaite et al., 2013; Dawson et al., 2016).

An alternative approach to SCR/Minute is the Integrated Skin Conductance Response (ISCR) (Benedek & Kaernbach, 2010). ISCR takes into account both the frequency and intensity of SCRs by calculating the area under the phasic EDA signal. This method offers the advantage of being continuous rather than simply an accumulation of SCRs.

### 1.1.3 Respiration

Movement artefacts, especially during position changes (standing up or sitting down), can impact the respiration signal. Bandpass filtering helps remove unwanted data (noise unrelated to breathing) by adjusting the filter frequencies based on the expected breathing rate. For example, an IIR bandpass filter set between 0.1 Hz and 0.6 Hz would correspond to a breathing rate of 6 to 36 breaths per minute. Normal breathing rate (eupnea) for healthy adults in rest typically range between 12 and 16 cycles per minute (Biopac, 2006; Stern, Ray, & Quigley, 2001), but values between 8-20 have also

been noted as normal (Brashers & Huether, 2014; Yuan, Drost, & McIvor, 2013). Compared to stationary subjects, a higher breathing rate is to be expected while participants are moving. Breathing rate also varies with age, and is higher for children, especially infants where it is normal with 40 breaths per minute or more (Fleming et al., 2011).

### 1.1.4 Brain activity

Our fNIRS studies using the Artinis Octamon system and Oxysoft software have primarily focused on the prefrontal cortex. In outdoor environments, sunlight and movement can introduce significant artifacts, particularly when combined. To mitigate this, participants should wear a dark cap to reduce light interference. This may also have the added benefit of making the fNIRS device less conspicuous.

fNIRS data inherently contains various noise sources. Pre-processing techniques like frequency filters, smoothing algorithms and wavelet filtering can improve data quality by removing physiological noise and artefacts (Dans, Foglia, & Nelson, 2021). Appropriate parameters should be set depending on the specificities of the experiment. As an example, it has been suggested that a bandpass filter between 0.1 and 0.4Hz is fruitful to remove physiological noise without interfering with the fNirs signal during a 10s task (Naseer & Hong, 2015). (A frequency of 0.4 Hz corresponds to 24 beats per minutes, thus removing e.g. the pulse frequency effectively). To address noise from extracerebral tissue reflections, short separation channels can be used for specific signal compensation (Zhou, Sobczak, McKay, & Litovsky, 2020).

For statistical analysis in Sensola, we have relied solely on block averaging, comparing average brain activity across different task block (Dale & Buckner, 1997).

### 1.1.5 Eye movements and pupilometry

To ensure good data quality, proper eye tracker positioning is crucial. This includes adjusting the glasses (top frame just above eyebrows, eyes cantered vertically) and ensuring a snug fit (Argus, 2023). Prescription glasses can be worn if they are well-positioned with the lower frames roughly aligned with the ETVision frames. Clean lenses are also important to avoid data artefacts.

We have encountered some instances of calibration drift during data collection, especially at the beginning of a session. This can be mitigated by comfortably securing the glasses before calibration. Performing a second calibration after a short period of time with the glasses on can further improve stability. Additional calibration checks throughout the experiment can be implemented for continued accuracy.

Our eye tracking data primarily focuses on fixation count, fixation duration, pupil diameter, and Areas of Interest (AOIs). We've consistently used standard ETVision and ETAnalysis settings and algorithms for data definition. Data quality has been good overall, with occasional calibration issues requiring data exclusion.

**References**

Argus. (2023). *ETVision Instruction Manual* Retrieved from Tyngsborough, MA USA: https://www.argusscience.com/file/ETVisionManual.pdf

Benedek, M., & Kaernbach, C. (2010). A continuous measure of phasic electrodermal activity. *J Neurosci Methods, 190*(1), 80-91. doi:10.1016/j.jneumeth.2010.04.028

Berntson, G. G., Quigley, K. S., Norman, G. J., & Lozano, D. L. (2017). Cardiovascular psychophysiology. In *Handbook of psychophysiology, 4th ed.* (pp. 183-216). New York, NY, US: Cambridge University Press.

Biopac. (2006). *Biopac Science Lab: Lesson 12 Respiration 1* Retrieved from Goleta, CA USA: https://www.biopac.com/wp-content/uploads/s12.pdf

Boucsein, W., Fowles, D. C., Grimnes, S., Ben-Shakhar, G., roth, W. T., Dawson, M. E., & Filion, D. L. (2012). Publication recommendations for electrodermal measurements. *Psychophysiology, 49*(8), 1017-1034. doi:10.1111/j.1469-8986.2012.01384.x

Braithwaite, J. J., Watson, D. P. Z., Jones, R. O., & Rowe, M. A. (2013). *Guide for Analysing Electrodermal Activity & Skin Conductance Responses for Psychological Experiments*. Retrieved from Birmingham, UK: https://www.birmingham.ac.uk/documents/college-les/psych/saal/guide-electrodermal-activity.pdf

Brashers, V. L., & Huether, S. E. (2014). Alterations of Pulmonary Function. In K. L. McCance & S. E. Huether (Eds.), *Pathophysiology : the biologic basis for disease in adults and children*. St. Louis, Missouri: Elsevier.

Dale, A. M., & Buckner, R. L. (1997). Selective averaging of rapidly presented individual trials using fMRI. *Hum Brain Mapp, 5*(5), 329-340. doi:10.1002/(sici)1097-0193(1997)5:5<329::Aid-hbm1>3.0.Co;2-5

Dans, P. W., Foglia, S. D., & Nelson, A. J. (2021). Data Processing in Functional Near-Infrared Spectroscopy (fNIRS) Motor Control Research. *Brain Sci, 11*(5). doi:10.3390/brainsci11050606

Dawson, M. E., Schell, A. M., & Filion, D. L. (2016). The Electrodermal System. In G. G. Berntson, J. T. Cacioppo, & L. G. Tassinary (Eds.), *Handbook of Psychophysiology* (4 ed., pp. 217-243). Cambridge: Cambridge University Press.

Fleming, S., Thompson, M., Stevens, R., Heneghan, C., Plüddemann, A., Maconochie, I., . . . Mant, D. (2011). Normal ranges of heart rate and respiratory rate in children from birth to 18 years of age: a systematic review of observational studies. *The Lancet, 377*(9770), 1011-1018. doi:10.1016/S0140-6736(10)62226-X

Pecchia, L., Castaldo, R., Montesinos, L., & Melillo, P. (2018). Are ultra-short heart rate variability features good surrogates of short-term ones? State-of-the-art review and recommendations. *Healthc Technol Lett, 5*(3), 94-100. doi:10.1049/htl.2017.0090

Quintana, D. S., Alvares, G. A., & Heathers, J. A. J. (2016). Guidelines for Reporting Articles on Psychiatry and Heart rate variability (GRAPH): recommendations to advance research communication. *Translational Psychiatry, 6*(5), e803-e803. doi:10.1038/tp.2016.73

Rohrbaugh, J. W. (2016). Ambulatory and Non-Contact Recording Methods. In G. G. Berntson, J. T. Cacioppo, & L. G. Tassinary (Eds.), *Handbook of Psychophysiology* (4 ed., pp. 300-338). Cambridge: Cambridge University Press.

Stern, R. M., Ray, W. J., & Quigley, K. S. (2001). *Psychophysiological recording*. New York: Oxford University Press.

Task Force, Malik, M., Bigger, J. T., Camm, A. J., Kleiger, R. E., Malliani, A., . . . Schwartz, P. J. (1996). Heart rate variability: Standards of measurement, physiological interpretation, and clinical use. *European Heart Journal, 17*(3), 354-381. doi:10.1093/oxfordjournals.eurheartj.a014868

Xie, R., Li, Q., Teng, L., Cao, Z., Han, F., Tian, Q., . . . Liu, Z. (2022). Strenuous exercise-tolerance stretchable dry electrodes for continuous multi-channel electrophysiological monitoring. *npj Flexible Electronics, 6*(1), 75. doi:10.1038/s41528-022-00209-0

Yuan, G., Drost, N. A., & McIvor, R. A. (2013). Respiratory rate and breathing pattern. *McMaster Univ. Med. J, 10*(1), 23-25.

Zhou, X., Sobczak, G., McKay, C. M., & Litovsky, R. Y. (2020). Comparing fNIRS signal qualities between approaches with and without short channels. *PLoS One, 15*(12), e0244186. doi:10.1371/journal.pone.0244186

Cerwén G and Hägerhäll CM (2025) Psychophysiological research in real-world environments: methodological perspectives from the SLU Multisensory Outdoor Laboratory. *Front. Psychol.* 16:1432180. doi: 10.3389/fpsyg.2025.1432180
